# Ambulatory blood pressure monitoring in children: A retrospective single-center study

**DOI:** 10.3389/fped.2023.1088857

**Published:** 2023-01-27

**Authors:** Mark Ahlenius, Wouter Koek, Ikuyo Yamaguchi

**Affiliations:** ^1^ Department of Pediatrics, Brooke Army Medical Center, San Antonio, TX, United States; ^2^ Department of Cell Systems and Anatomy, The University of Texas Health Sciences Center at San Antonio, San Antonio, TX, United States; ^3^ Division of Pediatric Nephrology, Department of Pediatrics, The University of Texas Health Sciences Center at San Antonio, San Antonio, TX, United States; ^4^ Division of Pediatric Nephrology and Hypertension, Department of Pediatrics, The University of Oklahoma Health Sciences Center, and Oklahoma Children’s Hospital, OU Health, Oklahoma, OK, United States

**Keywords:** pediatric primary hypertension, elevated blood pressure, ambulatory blood pressure monitoring (ABPM), left ventricular mass index (LVMI), left ventricular hypertrophy (LVH), cardiovascular disease

## Abstract

**Objectives:**

(1) Compare 24-hour ambulatory blood pressure monitoring (ABPM) diagnoses in a pediatric population with the new 2022 guidelines to the original diagnoses with the 2014 guidelines. (2) Determine whether findings of hypertension from ABPM could be predicted from prior patient data. (3) Determine whether ABPM readings could predict left ventricular mass index (LVMI) in patients who obtained an echocardiogram (ECHO).

**Study design:**

Single-center retrospective study on patients referred to Pediatric Nephrology Clinic for evaluation of elevated blood pressure who underwent ABPM from 2015 to 2018. Predictions of hypertension were obtained using a logistic regression model, and predictions of LVMI were performed using regression models including (a) the wake systolic and diastolic BP indices, or (b) additionally including the standard deviation (SD) of wake SBP and DBP.

**Results:**

With the change in 2022 to new ABPM guidelines from the AHA, comparing the old and new guidelines led to 70% of previous pre-hypertensive diagnoses now meeting criteria for diagnosis of hypertension, and a rise from 21% of the ABPMs meeting criteria for hypertension to 51% now meeting criteria. In a logistic regression model, prior patient data were not predictive of a diagnosis of hypertension from ABPM (Nagelkerke's *R*^2^ = 0.04). Among the individual variables studied, none were statistically significant. For prediction of LVMI, the SD of wake SBP and DBP were significantly associated with increased LVMI, but the wake SBP and DBP indices were not.

**Conclusions:**

In our patient population, the new ABPM guidelines led to a significant increase in diagnoses of hypertension. Prior patient data was not sufficient to predict a diagnosis of hypertension by ABPM, supporting the need for evaluation by ABPM as the gold standard. Our analysis of the relationship between ABPM readings and LVMI supports the hypothesis that BP variability contributes to increased LVMI. These data are consistent with growing evidence in the adult literature that BP variability detected by ABPM is associated with left-ventricular hypertrophy

## Introduction

Cardiovascular disease is the leading cause of death worldwide and accounts for 1 in 3 deaths in the United States (1). The prevalence of hypertension and elevated blood pressure in childhood is increasing, and target organ damage including cardiovascular changes has been found even in young children (2, 3). It is more important than ever before to identify hypertension in childhood and implement appropriate lifestyle changes and medications. In 2017, the American Academy of Pediatrics issued clinical practice guidelines for screening and management of high blood pressure in children and adolescents (AAP CPG) (4). 24-hour ambulatory blood pressure monitoring (ABPM) has become an important diagnostic tool in the evaluation and management of hypertension and has been used increasingly in clinic. However, the availability is still limited in pediatric clinics, and ABPM is usually provided by pediatric subspecialties such as pediatric nephrology or pediatric cardiology.

AAP CPG recommends ABPM should be performed to confirm the diagnosis of hypertension for patients with elevated BP for 1 year or more and patients with stage-1 HTN over 3 clinic visits. ABPM is more reliable and reproducible than clinic BP readings for the diagnosis of hypertension (4). Several studies have investigated whether other clinical data may help to determine the application of ABPM. A recent study from SHIP AHOY (Study of Hypertension In Pediatrics, Adult Hypertension Onset in Youth) found that office systolic BP above the 85th percentile for age, height, and gender based on repeated measurements is the optimal threshold for proceeding with ABPM (5). These guidelines were revised in 2022 by the AHA, changing the possible diagnoses to either normal or hypertensive (6). BP loads were eliminated from the analysis, and pre-hypertension is no longer a diagnosis.

The main purpose of BP diagnostics is to establish effective control to prevent the development of target organ damage. Left-ventricular hypertrophy (LVH) is a marker of target organ damage associated with hypertension and is an independent risk factor for cardiovascular morbidity and mortality in adults. Because the incidence of cardiovascular disease (CVD) in children and adolescents is low, LVH is used a surrogate marker for the risk of CVD in children. Previous studies have found that the BP data from ABPM could predict LVH better than clinic BP readings (7), and increased systolic BP measured by ABPM has been reported to increase the risk of LVH (7–11).

Other studies suggest that BP variability (BPV) may play a key role in LVH and decreased LV function (12–14). BPV can be long-term visit-to-visit variation of office BP measurements, mid-term day-to-day variation of home BP monitoring, or short-term hour-to-hour variation measured by ABPM (15). BPV indices that have been used include standard deviation (SD), coefficient of variation (CV), variability independent of the mean (VIM), and average real variability (ARV) (16). SD is the measure most commonly used for studies of BPV effects on CVD outcomes. Boubouchairopoulou et al. (17) assessed BPV among office, home, and ambulatory BP readings with SD, CV, and VIM. They found that detection of individuals with high BPV was affected by the methods of BP measurement, but the choice of BPV index was less important. Stevens et al. (18) reported a systematic review and meta-analysis of BPV and cardiovascular disease. Most data used SD as the measure of BP variability. They found that mid-term and short-term variability of daytime systolic blood pressure were associated with risk of cardiovascular disease events and mortality.

In the present study, we collected data from the patients who were referred to our pediatric nephrology clinic at University Hospital System and the University of Texas Health Sciences Center at San Antonio for elevated blood pressure and underwent ABPM from 2015 to 2018.

The goals of this study were to (1) Determine the effects of changes in the guidelines on ABPM diagnoses. (2) Determine whether findings of hypertension from 24-hour ABPM could be predicted from prior patient data, and (3) Determine whether ABPM data including the wake and sleep mean BP and BPV could predict left ventricular mass index (LVMI) in patients who obtained an echocardiogram (ECHO).

## Materials and methods

### Subject selection and data source

This is a retrospective chart review. The patients enrolled were seen in our pediatric nephrology clinic for elevated blood pressure or hypertension and underwent a 24-hour ABPM from February 2015 to August 2018. Clinical data, including demographics, clinical presentation, medical history, comorbidities, ABPM results, family history, and laboratory values were obtained by chart review. Exclusion criteria included taking anti-hypertensive medications on initial ABPM and the following co-morbidities: chronic kidney disease, solid organ transplant, bone marrow transplant, congenital heart disease, malignancy, diabetes, and obstructive sleep apnea.

The Space Labs 90217 monitor (Issaquah, WA), validated in children, was used for all ABPM. The non-dominant arm was used, and patients were encouraged to attend school but avoid vigorous activity. Awake and sleep times were set according to patient report, and ABPM readings were taken every 20 min in the daytime and every 30 min at night. The adequacy of the ABPM study was determined by the interpreting physician at the time of ABPM evaluation according to AHA criteria, and the data were interpreted using American Heart Association (AHA) guidelines (19). Retrospective data collection was approved by the Institutional Review Board at the University of Texas Health Science San Antonio and the University Hospital System.

### BP and ABPM analysis

The office BP readings were taken when ABPM was placed in clinic. These readings were evaluated based on 2017 AAP clinical practice guidelines (4). BP percentiles greater than or equal to 90 were interpreted as abnormal for the patients with ages less than 13 years, and greater or equal to 120/80 were interpreted as abnormal for the patients with ages greater than or equal to 13 years.

The initial diagnosis of the ABPM study was done by 2014 AHA ABPM guidelines (19). Briefly, the ABPM study reports included mean and standard deviation (SD) of awake systolic BP readings, awake diastolic BP readings, sleep systolic BP readings, and sleep diastolic BP readings. Each reading was converted to an index by dividing by the threshold, which was determined as the 95th percentile of the ABPM reference data based on gender and height. The BP loads were determined as the percentage of BP readings greater than or equal to the threshold. Hypertension was defined by a mean ABPM index greater than or equal to 1 for any of the four types of readings. Pre-hypertension was defined by all mean ABPM indices less than 1, but BP load greater than or equal to 25 percent for any of the four types of readings. Normal BP was defined by all mean ABPM indices less than 1 and all BP loads less than 25 percent. In this paper, we categorized ABPM results as either normal or hypertension, where normal included the original diagnoses of normal and pre-hypertension. These analyses were then compared to the new criteria of the 2022 AHA ABPM guidelines (6). For the criteria of hypertension for adolescents 13 years of age or older, the mean 24-hour BP is greater than 125/75, the mean wake BP is greater than 130/80, or the mean sleep BP is greater than 110/65. The criterion for hypertension in children less than 13 years of age is mean BP greater than or equal to 95th percentile (6). Based on the studies described in the Introduction (17, 18), BPV was assessed by the SD of wake SBP and wake DBP.

### Echo study

Echo was done by physician's discretion by primary providers, pediatric cardiologists, or pediatric nephrologists. A comprehensive 2-dimensional Doppler and M-mode echo was performed, and left ventricular mass (in grams) was calculated by the Devereux et al. method (20). The left ventricular mass was indexed to height (in m^2.7^) to obtain the LVMI. LVMI was normalized by age-specific 95th percentile thresholds (21). LVH was reported by two definitions: LV mass >51 g/m^2.7^ height for subjects older than 8 years and LVMI > 95th percentile (21) for all subjects.

### Statistical analysis

Potential predictors of the binary outcome variable hypertension were analyzed individually using univariable logistic regression and simultaneously using multivariable logistic regression. The statistical significance of the effect of each predictor was evaluated by Wald tests, and the magnitude of the effect was expressed as the odds ratio and its 95% confidence limits. Potential predictors of LVMI were analyzed individually and simultaneously by linear regression, which was performed separately for index variables only and for index and SD variables together; the two different models were compared with ANOVA. All statistical analyses were conducted using R implemented in jamovi Version 2.3 (The jamovi project, 2022; https://www.jamovi.org.)

## Results

### Changes in diagnosis based on the new ABPM guidelines

**Figure 1:**
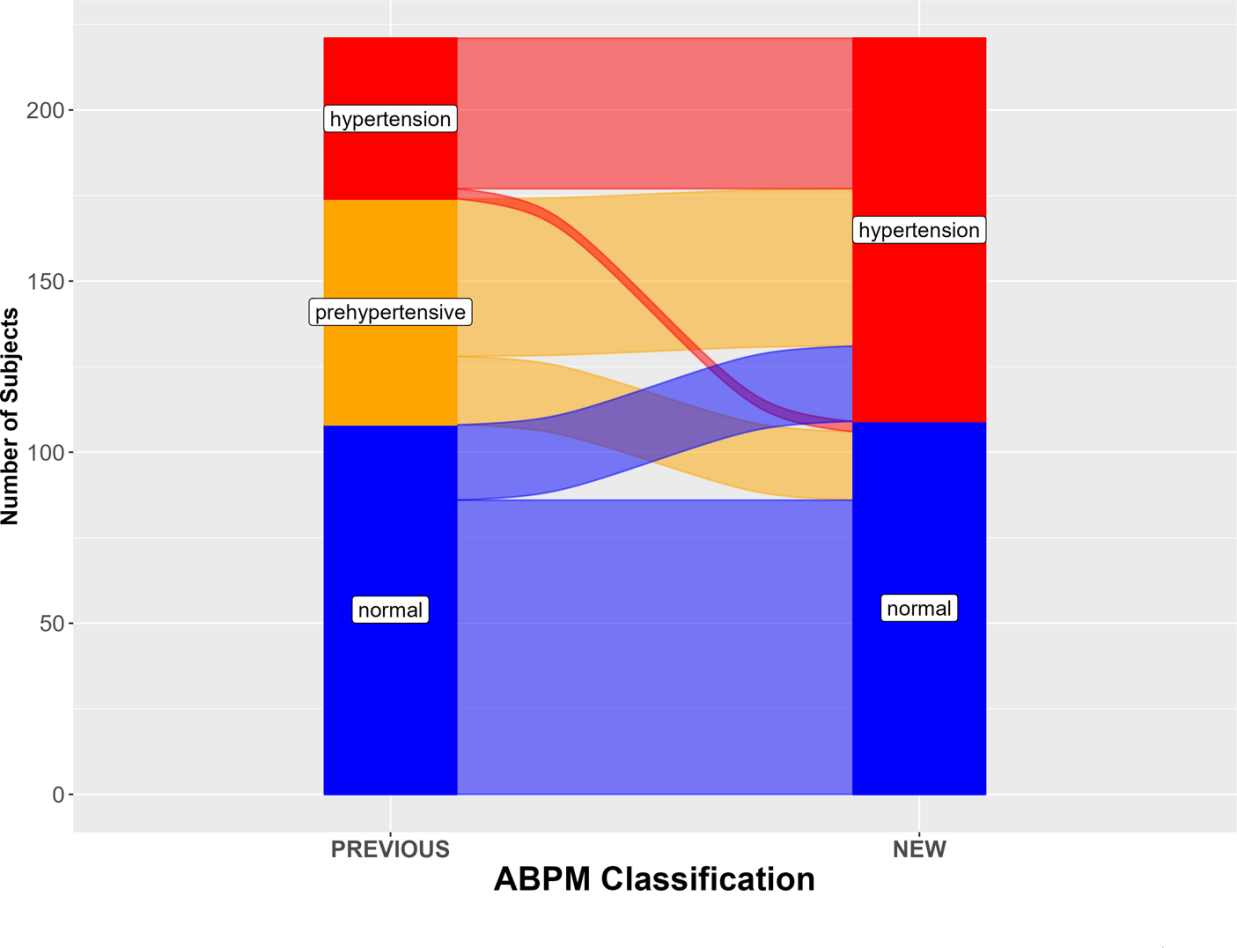
ABPM diagnoses according to new (2022) and previous guidelines, in 221 subjects. The alluvial plot is based on the values reported in Table 2.

This study included data from 221 subjects that satisfied inclusion criteria. Subjects were 5–20 years of age when ABPM data was collected. Only 9 subjects (2.3%) failed ABPM. The average number of readings was 52, 69% of the readings were successful, and the average number of hours was 24. Table 1 shows the characteristics of the 221 subjects. The mean age was 14.1 years. 80.5% were Hispanic, reflecting the demographics of the patients seen in our center, and 72.9% were male. The mean BMI Z-score was 1.8, suggesting that the subjects were mostly overweight or obese (Table 1). The initial ABPM diagnoses in 221 subjects were normal in 108 subjects (49%), pre-hypertension in 66 subjects (30%), and sustained hypertension in 47 subjects (21%). Under the new criteria, the ABPM diagnoses were normal in 109 (49%) and hypertension in 112 (51%). 20% of the previous normal ABPM diagnoses now met criteria for hypertension, and 70% of the pre-hypertension subjects met criteria for hypertension (Table 2 and Figure 1). The overall prevalence of nocturnal hypertension in our cohort was 42.5% (94 subjects out of 221, including 46 subjects with only nocturnal hypertension).

**Table 1:** Patient demographics (*n* = 221).

Variable	Mean ± SD or Number (%)
Age, years	14.08 ± 2.84
Gender, Male, %	72.9
Ethnicity, %	
Hispanic or Latino	80.5
NOT Hispanic or Latino	18.6
Unknown / Not Reported	0.9
Race, %	
White	89.6
Black or African American	6.3
American Indian/Alaska Native	1.4
Height, cm	162.48 ± 15.03
BMI, kg/m^2^	29.24 ± 7.24
BMI z-score	1.8 ± 1.0
ADHD, %	18.6

BMI, body mass index; ADHD, attention deficit hyperactivity disorder.

**Table 2:** ABPM diagnoses by the new 2022 criteria vs. the old criteria.

Previous ABPM classification	New 2022 ABPM classification		
** **	Normal	Hypertension	Total
Normal (%)	86 (80)	22 (20)	108 (100)
Pre-hypertensive (%)	20 (30)	46 (70)	66 (100)
Hypertension (%)	3 (6)	44 (94)	47 (100)
Total (%)	109 (49)	112 (51)	221 (100)

### Was the ABPM diagnosis predictable?

We examined the ability of prior patient data including gender, age, BMI, family history of hypertension, office BP reading, and a diagnosis of ADHD to predict the ABPM results. The outcome variable investigated was hypertension status: hypertensive or normal. The variables shown in Table 3 are potential predictors of the outcome variable. Binomial logistic regression was used. Overall, the regression model did not predict hypertension status (Nagelkerke's *R*^2^ = 0.04; *χ*^2^ = 6.56, *df* = 7, *p* = 0.47). Among the individual predictor variables, only male gender neared statistical significance (*p* = 0.09). Obesity and the family history of hypertension were not associated with hypertension in our cohort, although previous studies have found an association (19, 22). The diagnosis of attention deficit hyperactivity disorder (ADHD) had no significant relationship to the diagnosis of hypertension. Overall, these data indicate that the ABPM diagnosis was not predicted by the prior patient data, supporting the use of ABPM in all patients with elevated BP readings, irrespective of the predictor variables investigated here.

**Table 3:** Model coefficients for logistic regression analysis predicting hypertension.

	Multivariable					Univariable	
Predictor	Estimate	SE	Z	*p*	OR (95% CI)	OR (95% CI)	*p*
Intercept	0.57	0.87	0.66	0.507	1.77 (0.33–9.67)		
Gender: Female-Male	0.54	0.32	1.68	0.092	1.72 (0.91–3.22)	1.81 (0.99–3.30)	0.054
Age	−0.02	0.05	−0.40	0.688	0.98 (0.89–1.08)	0.97 (0.88–1.06)	0.486
BMI (Z score)	−0.01	0.14	−0.08	0.933	0.99 (0.74–1.31)	0.99 (0.75–1.30)	0.930
Family history of hypertension: yes/no	−0.21	0.31	−0.67	0.504	0.81 (0.45–1.49)	0.84 (0.47–1.51)	0.566
Systolic percent ≥90	−0.27	0.31	−0.88	0.380	0.76 (0.41–1.40)	0.68 (0.38–1.21)	0.188
Diastolic percent ≥90	−0.24	0.33	−0.73	0.465	0.78 (0.41–1.50)	0.77 (0.41–1.43)	0.401
ADHD: yes/no	−0.30	0.37	−0.80	0.424	0.74 (0.36–1.53)	0.77 (0.39–1.51)	0.441

Multivariable: all predictors analyzed together. Univariable: each predictor analyzed separately.

BMI, body mass index; ADHD, attention deficit hyperactivity disorder; SE, standard error; OR, odds ratio; CI, confidence interval.

### Could ABPM readings predict left ventricular mass index (LVMI)?

Among the 221 subjects, 40 had an echocardiogram (ECHO). Their LVMI values varied between 25.0 and 79.7 g/m^2.7^ with a mean of 40.6 g/m^2.7^ and a standard deviation (SD) of 11.0 g/m^2.7^. Fourteen of the 40 subjects were found to have LVH based on LVMI > 95th percentile (21), and 2 out of 37 subjects older than 8 years were found to have LVH based on the adult criterion of >51 g/m^2.7^ height by the AAP CPG criteria. All but one of the 14 subjects with LVH were obese. Ten of these subjects were diagnosed with hypertension, and the other 4 were diagnosed as normal.

For our analysis, the LVMI value normalized by the 95th percentile (17) was used as the dependent variable. Two multiple linear regression models were used to investigate whether the LVMI could be predicted from the ABPM data (Table 4). In Model 1, the predictor variables were the two mean BP indices (systolic, diastolic). Model 2 included both the two mean BP indices and the SD of each BP index. The adjusted *R^2^* for Model 2 (0.2250) was higher than the value for Model 1 (0.0943), but the difference was not significant (F[2,35] = 2.95, *p* = 0.065. Neither model provided a statistically significant prediction of LVMI (*p* = 0.057 for Model 2). However, univariable analyses showed that both the systolic SD and the diastolic SD were significantly associated with LVMI (*p* = 0.034 and 0.049, respectively)), but the index variables were not (*p* = 0.508; *p* = 0.225) (Table 4). LVMI did not differ significantly between hypertensive subjects with and without nocturnal hypertension, 38.49 ± 7.68 (g/m^2.7^) vs. 38.50 ± 7.95 (g/m^2.7^), respectively.

**Table 4:** Model coefficients for linear regression analysis predicting LVMI (g/m^2.7^).

Model 1							
** **	Multivariable	** **	** **			Univariable	** **
Predictor	Estimate	SE	*t*	*p*	95% CI	Estimate (95% CI)	*p*
Intercept	1.010	0.450	2.246	0.031	0.099–1.921		
Systolic Index	0.756	0.501	1.509	0.140	−0.259–1.771	0.30 (−0.608–1.208)	0.508
Diastolic Index	−0.856	0.466	−1.837	0.074	−1.80–0.088	−0.507 (−1.340–0.325)	0.225
Model 2							
** **	Multivariable					Univariable	
Predictor	Estimate	SE	*t*	*p*	95% CI	Estimate (95% CI)	*p*
Intercept	0.862	0.433	1.992	0.054	−0.016–1.741		
Systolic Index	0.449	0.493	0.910	0.369	−0.552–1.450	0.30 (−0.608–1.208)	0.508
Diastolic Index	−0.830	0.443	−1.875	0.069	−1.730–0.069	−0.507 (−1.340–0.325)	0.225
Systolic SD	0.019	0.013	1.444	0.158	−0.008–0.046	0.026 (0.002–0.051)	0.034
Diastolic SD	0.022	0.018	1.210	0.234	−0.015–0.058	0.033 (0.0001–0.067)	0.049

Multivariable: all predictors analyzed together (Shapiro-Wilk normality test *p* = 0.22). Univariable: each predictor analyzed separately.

BMI, body mass index; ADHD, attention deficit hyperactivity disorder; SD, standard deviation; SE, standard error; CI, confidence interval.

## Discussion

Our study presents data from a large cohort of children who were referred for elevated blood pressure to a single pediatric nephrology clinic and underwent ABPM. With the revised classification of ABPM in 2022 by the AHA, we found a significant increase in diagnoses of hypertension, including children previously diagnosed as either normal or pre-hypertension. This is expected, given lower thresholds for mean awake BP and mean sleep BP readings, particularly in tall males. This was found in our cohort. 70 percent of previously pre-hypertensive subjects were found to be hypertensive by 2022 ABPM guidelines, and 30 percent were found to be normotensive. These pre-HTN to normotensive subjects included a larger percentage of females and were shorter than the pre-HTN to hypertensive subjects.

The study was focused on subjects without co-morbidities to identify primary hypertension and estimate the risk of target organ damage with the new ABPM criteria. Our results showed that the presence of sustained hypertension was not predicted by the patient data available prior to ABPM. We found that clinic BP readings were poorly correlated with the diagnosis of hypertension. All subjects were referred for elevated BP on multiple visits in clinic, but only 21% were found to have hypertension by ABPM at the time of diagnosis. Even under the new guidelines, only 51% were found to be hypertensive. Almost half of the subjects were found to have white-coat hypertension, which is consistent with other studies (22–24). Davis et al. (25) compared among clinic SBP, clinic SBP in combination with ABPM, or ABPM alone for children, concluding that universal ABPM may be the most economical and effective diagnostic strategy. They recommended universal ABPM in children who were referred for elevated BP. In contrast, the results of the SHIP AHOY study suggested that clinic systolic BP of 85th percentile based on the AAP CPG reference data may be the optimal threshold to perform ABPM (5). Johnson et al. (26) suggested that normal auscultative systolic BP in clinic weakly predicted normal ABPM in children. Our study did not find that clinic SBP readings help to predict the diagnosis of hypertension.

Why do clinic BP readings fail to predict the results of ABPM? One reason is that clinic BP data are seldom obtained in the recommended manner prior to referral for ABPM. Rea et al. found that only 2% of patients had followed the recommended steps from the 2017 AAP CPG for assessment of clinic BP readings, only 10% had a follow-up appointment, and 2% had recommended lab tests (27). ABPM is generally not available in primary care practice because of cost, poor reimbursement, and the time required for data collection and interpretation. This barrier to accurate identification of HTN may be overcome at least for children 13 years of age or older, as the definition of normal BP was simplified to be less than 120/80, which is consistent with the adult criterion. Increasing ABPM accessibility for children and adolescents in primary practice may be a better approach for BP screening. The other goal of this study was to investigate whether the ABPM data predict LVMI. Sustained hypertension is well known to increase left ventricular mass and cardiovascular events, including early-onset events (28). In adults, LVH defined by LVMI greater than 51 g/m^2.7^, predicts a 3.3 times higher risk of a CV event (29). Our study showed that increased wake SBP variability and DBP variability, not wake SBP index or wake DBP index, was the best predictor of increased LVMI. This is an interesting finding, as many studies have reported that SBP and/or SBP load predict LVMI or LVH (7, 10, 11, 30). BP variability has been reported to increase cardiovascular events in adults (12, 13, 31). Shin et al. investigated the relationship between daytime SBP variability measured by ABPM and LVMI. They found that higher daytime SBP variability and daytime mean SBP were associated with increased LVMI as well as increased arterial stiffness evaluated by pulse wave velocity and augmentation index (14). Richey et al. studied LVH in children and adolescents with primary hypertension, finding that subjects with LVH had increased 24-hour SBP standard deviation score (SDS) and 24-hour DBP SDS (8). Furthermore, improvement of LVMI after 3 years on anti-hypertensive medications was associated with reduced systolic and diastolic BP variability (32). In combination, our data and these previous findings suggest that BP variability is likely to be an important factor in the development of LVH.

The majority of our subjects with LVH were overweight or obese, and the total number of subjects was limited. For these reasons, the data were not sufficient to determine the relative effects of overweight/obesity vs. hypertension on LVMI. Previous studies showed that obesity and hypertension were both independently associated with LVH in children (10, 33).

### Limitations

Although the present study included a large number of ABPM recordings, they are all from a single center and reflect the standard practice at one institution, so generalizability may be limited, and referral bias may be present. The demographics of our patient population were somewhat different from those of many other centers in the United States, including a majority of the subjects identifying as Hispanic/Latino. The diagnosis of OSA was excluded in this cohort. However, we did not screen all patients for OSA, and there is a potential for undiagnosed OSA in this population.

We only used one office BP reading in this cohort, and the majority of the readings were obtained using oscillometric devices. Although auscultatory measurement is the gold standard, our clinic was not set up specifically for pediatric nephrology, and BP was not usually measured by auscultation. We did have previous blood pressure readings from prior to referral. However, the time differences between these were variable, and in many cases lifestyle modifications may have been initiated by the PCP while the subject was awaiting further evaluation. Also, we were unable to track how these previous BP readings were obtained, so these data appeared of limited value for our study. Echocardiograms were obtained by provider's discretion, not by a protocol or criteria, as most of the data were collected before the 2017 AAP Pediatric HTN guidelines, and the number of echo studies was limited. These factors may have limited our ability to discern the relationships between prior clinical data and the ABPM diagnosis, and between the ABPM data and LVMI.

## Conclusion

In our patient population, with the implementation of the new AHA ABPM criteria in 2022 we saw a rise in total diagnoses of hypertension in our patient population, with the most dramatic change being previously pre-hypertensive patients joining the hypertensive category. Prior patient data were not sufficient to predict a diagnosis of hypertension from ABPM, supporting the need for evaluation by ABPM as the gold standard. Our analysis of the relationship between ABPM readings and LVMI supports the hypothesis that systolic BP variability contributes to increased LVMI. These data are consistent with growing evidence in the adult literature that BP variability detected by ABPM is associated with left ventricular hypertrophy.

## Data Availability

The raw data supporting the conclusions of this article will be made available by the authors, without undue reservation.
